# Immunomodulation of the Tumor Microenvironment: Turn Foe Into Friend

**DOI:** 10.3389/fimmu.2018.02909

**Published:** 2018-12-11

**Authors:** Hanne Locy, Sven de Mey, Wout de Mey, Mark De Ridder, Kris Thielemans, Sarah K. Maenhout

**Affiliations:** ^1^Laboratory of Molecular and Cellular Therapy (LMCT), Vrije Universiteit Brussel, Brussels, Belgium; ^2^Department of Radiotherapy, UZ Brussel, Vrije Universiteit Brussel, Brussels, Belgium

**Keywords:** immunotherapy, oncolytic virotherapy, radiotherapy, cancer, *in situ* vaccination

## Abstract

Immunotherapy, where the patient's own immune system is exploited to eliminate tumor cells, has become one of the most prominent new cancer treatment options in the last decade. The main hurdle for classical cancer vaccines is the need to identify tumor- and patient specific antigens to include in the vaccine. Therefore, *in situ* vaccination represents an alternative and promising approach. This type of immunotherapy involves the direct intratumoral administration of different immunomodulatory agents and uses the tumor itself as the source of antigen. The ultimate aim is to convert an immunodormant tumor microenvironment into an immunostimulatory one, enabling the immune system to eradicate all tumor lesions in the body. In this review we will give an overview of different strategies, which can be exploited for the immunomodulation of the tumor microenvironment and their emerging role in the treatment of cancer patients.

## Introduction

Already in 1909, Paul Ehrlich postulated that the immune system has the ability to suppress the majority of carcinomas and thus plays an important role in the protection against tumor development (1). Instrumental to this idea is the capacity of the immune system to distinguish “self” from “non-self” and to eliminate the latter without damaging the former.

To pursue the specificity of immunotherapy, various efforts have been made to identify cancer-associated antigens to use in therapeutic vaccination strategies. The first tumor-associated antigens (TAAs) identification was made in the context of melanoma with melanoma antigen family A1 (MAGE-A1) identified in 1991 (2). MAGE-A1 is a member of a large gene family, comprising 25 cancer-germline genes. This identification was followed by the observation that T cells frequently target proteins associated with pigment production in melanomas (3). These tissue differentiation antigens, which are normal proteins with a specific function in the target tissue, constituted the majority of initially discovered TAAs. However, targeting these antigens can lead to severe, life threatening side effects due to expression of these antigens, even in low amounts, by normal tissue (4, 5). Tumors can also overexpress normal self-proteins, that are important for their malignant phenotype, such as p53 and human Telomerase Reverse Transcriptase (hTERT). Given the important role of these proteins for the survival and phenotype of cancer cells, tumors cannot downregulate these molecules and this makes them an attractive target for immunotherapy. However, since they have normal functions in some tissues and under certain conditions, off-tumor reactions can occur when targeting these proteins (6). In recent years, with the development of deep sequencing technologies, studies have revealed the presence of antigens resulting from somatic mutations and giving rise to proteins with altered sequence. These mutation-derived antigens, also known as neo-antigens, are tumor- and patient-specific. Targeting neo-antigens would overcome self-tolerance and lead to stronger immune responses (7, 8). Due to the heterogeneity within tumors and since cancer vaccines only target a limited number of antigens, cancer cells that do not express these antigens can escape immune control and give rise to new tumor populations that can resist treatment with a vaccine encoding the same TAAs (9). Moreover, T cells evoked after vaccination often fail to infiltrate in the tumor or fail to exert their function due to immunosuppression in the tumor (10).

With *in situ* vaccination these problems can be circumvented. *In situ* vaccination refers to any approach where the tumor vaccine antigens are processed in the patients own body following intratumoral (IT) treatment with immunostimulatory drugs. These immunomodulators have the capacity to stimulate tumor cell death and therefore enhance the uptake and presentation of TAAs by APCs. With this strategy, the need to identify TAAs to include in the vaccine is circumvented thereby limiting labor-, time-, and cost-intensive *ex vivo* efforts. The generation of anti-tumor T cells at one tumor site should allow them to attack distant tumor lesions resulting in a systemic immune response. Moreover, since *in situ* vaccination depends on the local injection of immunostimulatory molecules, systemic toxicities are limited (11). Overall, lower amounts of reagents are required when administered locally, significantly reducing the cost of therapies (e.g. for checkpoint inhibitors). Since *in situ* vaccination is not personalized but available off-the-shelf, this therapy can be combined with other standard of care treatments, such as surgery and radiotherapy, in order to find the most optimal treatment schedule resulting in curing the patient.

## *In situ* Vaccination: Activation of the Immune System

An *in situ* vaccine should be able to convert an immunosuppressive or dormant tumor microenvironment (TME) into an immunostimulatory one, which allows effector T cells to enter the tumor bed and to kill the tumor cells. Such an anti-tumor immune response will only lead to effective killing of cancer cells when a series of events occurs in a specific order, resulting in the proper activation of the immune system.

The innate immune response starts with the recognition of pathogens (characterized by Pathogen-Associated Molecular Patterns, PAMPs) or indicators of danger (Damage-Associated Molecular Patterns, DAMPs) by pathogen-recognition receptors (PRRs). Immature dendritic cells scan the periphery and when they encounter such a PAMP or DAMP, they efficiently take up antigens and undergo maturation under the influence of a number of danger signals, various cytokines and tissue factors. These DCs present antigens in the context of Major Histocompatibility Complex (MHC) class I and II molecules to activate both CD8^+^ and CD4^+^ T cells. Different activation signals are needed for a T cell before they can exert their function. The initial interaction between the DC and the T cell, through the MHC complex and the T cell receptor, provides the first signal. A so-called second signal concerns a costimulatory interaction between CD28 on T cells and CD80 or CD86 on APCs, and is also required for T cell activation. CD8^+^ T cells also require additional cytokine signals (signal 3), for the optimal generation of effector and memory populations and for their survival (12, 13). The absence of these signals and the presence of immunosuppressive cytokines could either activate T helper 2 cells or attract and activate regulatory T cells (Tregs), myeloid-derived suppressor cells (MDSCs) or dysfunctional DCs leading to immunosuppression (14). Tumors can increase the production of immunosuppressive cytokines, reduce the expression levels of MHC I molecules, downregulate their expression of TAAs, thereby evading immune recognition and eventually escape immune control.

With *in situ* vaccination, changes in cytokine secretion patterns are induced, leading to changes in the type, number and activation status of tumor-infiltrating lymphocytes (TILs), resulting in an effective anti-tumor immune response (15, 16). A second important feature of an *in situ* vaccine is the ability to induce immunogenic cell death (ICD). ICD is defined as a specific form of regulated cell death that induces the release of TAAs and triggers an anti-tumor immune response (17). During ICD, there is a timely release of DAMPs that warns the organism of a situation of danger, resulting in the induction of an immune response associated with the formation of an immunological memory. Although ICD is a very complex process, six DAMPs are mechanistically linked to the induction of this type of cell death and the subsequent immune response. Firstly there is calreticulin (CRT), an ER-associated chaperone protein that promotes phagocytosis of dying cells by attracting DCs (18). The second DAMP is high mobility group box 1 (HMGB1), a histone-chromatin binding protein passively released from stressed or dying cells. HMGB1 exerts potent immunomodulatory effects by binding to Toll Like Receptor (TLR) 4 and TLR9, which both play crucial roles in driving inflammatory responses (19). Extracellular ATP is the third DAMP, that is sensed by the purinergic receptor P2X7, a key regulatory element of the inflammasome, leading to the secretion of pro-inflammatory cytokines resulting in the attraction of DCs toward the dying tumor cells (19–22). The fourth DAMP is type I IFN, which is produced by cancer cells undergoing ICD in response to endogenous double stranded (ds) RNA detected via TLR3 (23) or in response to dsDNA sensed by cGAS (24–26). Type I IFN mediates various immunostimulatory effects on immune cells (27). Cancer cell-derived nucleic acids are the fifth DAMP that play a role in ICD. Cancer cell-derived nucleic acids are taken up by DCs, neutrophils and macrophages, resulting in a potent type I IFN response (28–31). Lastly there is extracellular ANXA1, which supports the activation of adaptive immune response by engaging formyl peptide receptor 1 (FPR1) on DCs (32). All these DAMPs play a role in the outcome of ICD and will determine the strength and the durability of the anti-tumor responses.

**Figure 1:**
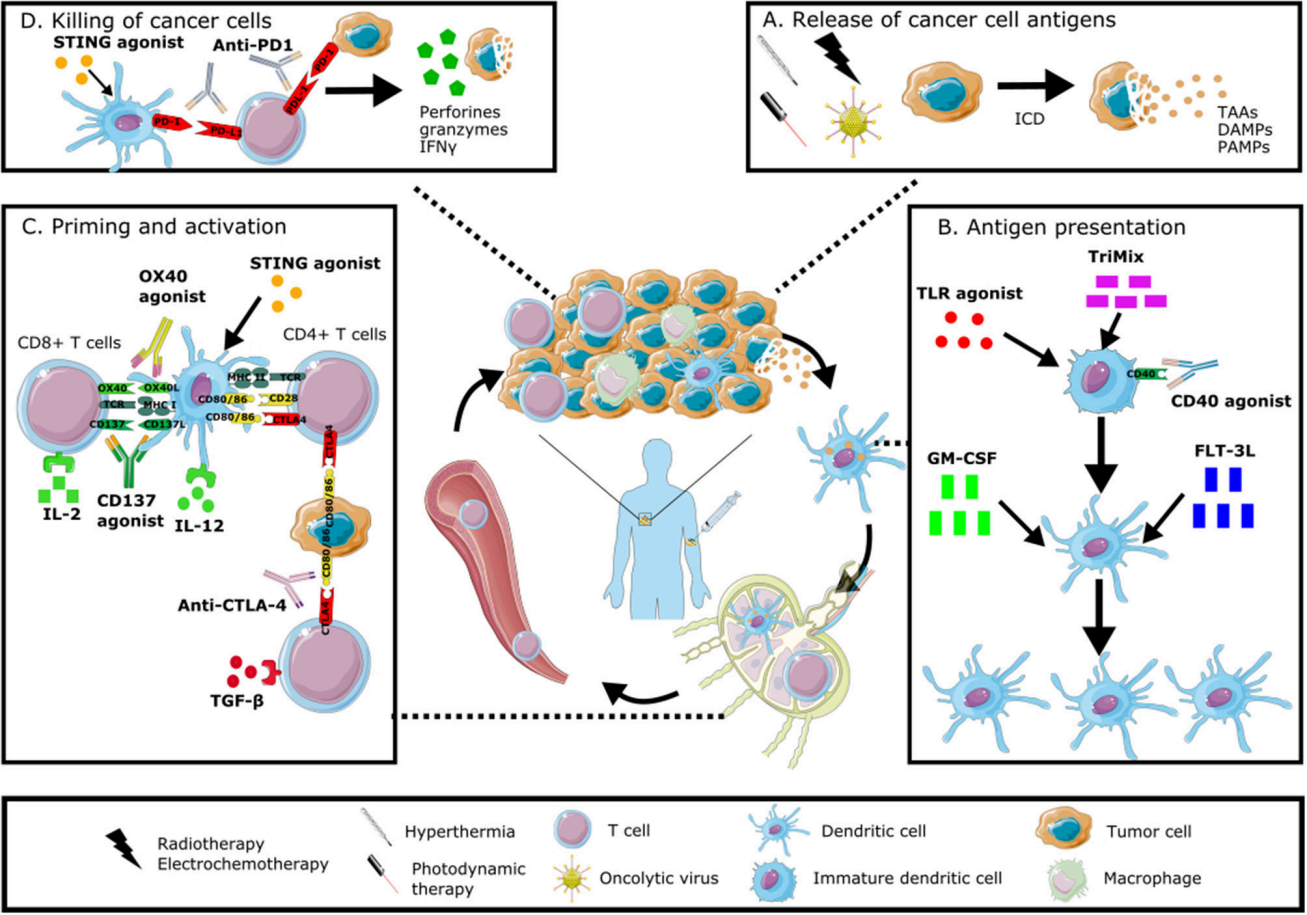
Immunomodulation of the tumor microenvironment to induce anti-tumor immune responses. *In situ* vaccines result in intratumoral modulation to attract and activate dendritic cells able to present the full antigenic repertoire to tumor-specific T cells able to kill tumor cells. This immunomodulation can occur at different levels: stimulating the induction of immunogenic cell death with radiotherapy, electrochemotherapy, hyperthermia, photodynamic therapy or oncolytic viruses **(A)**, increasing the number and maturation of dendritic cells through the administration of growth factors, cytokines or TLR agonists **(B)**, stimulating the priming and activation of T cells through the intratumoral injection of checkpoint inhibitors, cytokines or other immunomodulating agents **(C)**, promoting the direct killing of cancer cells through the local administration of STING agonists or checkpoint inhibitors **(D)**. All of these modalities can be combined in order to induce a robust anti-tumor immune response. Graphical elements are adapted from Servier medical art repository (https://smart.servier.com).

In this review we will discuss preclinical and clinical data of different *in situ* vaccination strategies that stimulate anti-tumor immune responses through the induction of ICD, the attraction of different immune cell populations and by alleviating immune suppression. The discussed immunomodulators include oncolytic viruses, radiotherapy, physical therapies, growth factors and cytokines, as well as combinations of these modalities. An overview of these modalities and their mechanism of action is given in Figure 1.

## Immunomodulatory Approaches: How to Make a Cold Tumor Hot?

### Oncolytic Viruses (OVs)

The interest in oncolytic virotherapy is not a new concept, but has grown exponentially during the last years alongside the advancements in molecular biology, virology, immunology and genetic engineering (33).

Oncolytic viruses (OVs) are attenuated, mutated, or benign viruses that preferentially target cancer cells and do not infect normal, non-transformed cells. The list of OVs used for therapy is rapidly growing and includes reovirus, vesicular stomatitis virus, vaccinia virus, Newcastle disease virus, measles virus, poliovirus, herpes simplex virus, coxsackievirus, adenovirus, and Maraba virus.

The anti-tumor effect of OVs arises from a dual mechanism of action: the selective replication of the virus in tumor cells will result in cell killing while simultaneously stimulating the immune system through the induction of ICD. Via the recruitment and activation of cross-presenting DCs followed by the stimulation of specific lymphocytes this ICD will induce an effective anti-tumor immune response (34). The key desirable characteristics of OVs are therefore the specificity for the targeted cancer cells, their potency to induce ICD and safety to avoid adverse reactions and pathogenic reversion (35). Numerous naturally occurring OVs exist, but recently immense interest has revolved around genetically modifying viruses to improve their safety, specificity, immunogenicity, oncolytic potency, and drugability (35). All clinical related OVs have been genetically modified with one or more immunomodulating agents (As described in the section Immunomodulatory factors).

#### Immune Modulation by OVs

Originally OVs were designed to be cytolytic agents, but it is now clear that they have pleiotropic effects on the TME through activation of different signaling pathways (36). Triggering of ICD in OV-infected cancer cells results in the release of PAMPs in the TME. Tumor cell derived PAMPs, for example viral capsids, DNA, RNA, and proteins, are important drivers of adjuvanticity and effective APC engagement, and are even more important than the mode of cell death (37, 38). The innate immune pathways and sensors that can be triggered by OVs induced PAMPS have been largely uncovered. This innate immune response is mainly mediated by a set of TLRs (expressed on the plasma membrane and in endosomal compartments), cytoplasmic receptors, and intracellular NOD-family of receptor complexes. The most important TLRs are TLR3/TLR7, which recognizes viral double stranded (ds) RNA and single stranded (ss) RNA and TLR9, which recognizes ss DNA. Upon infection of tumor cells with RNA/DNA-based OVs these TLRs may promote the intrinsic (in the tumor) and extrinsic (in the phagocyte) production of cytokines in the TME (39, 40). The cytoplasmic receptors Retinoic acid Inducible Gene 1 (RIG-I) and Melanoma Differentiation-Associated protein 5 (MDA-5) play a crucial role in the recognition of RNA from OVs. Both receptors can activate cytokine production through the mitochondrial antiviral signaling (MAVS) adaptor protein upon infection with OVs such as vesicular stomatitis virus (VSV) and measles viruses (40). In addition, it has become clear that innate immune STimulator of Interferon Genes (STING) signaling through the cGAS-STING complex plays a vital role in directing T cell responses toward infected tumor cells. After phagocytosis of the tumor cells, the partially degraded genomic DNA, which was compartmentalized in the nucleus, is efficiently processed by DNase II in the lysosomal compartment (41, 42). However, a small fraction of genomic DNA can leak out the lysosomal compartment resulting in activation of the STING pathway. Cyclic guanosine monophosphate-adenosine monophosphate synthase (cGAS), a cellular synthase, binds to these cytosolic nucleic acids, which generates self-DAMPS referred to as cyclic dinucleotides. At this point the cGAS-STING signaling complex is formed which triggers type I interferon (IFN) production required for cross-priming of TAAs and the generation of tumor specific T cells (43).

The intercellular transfer of a TAA released in the TME induced by different OVs upon infection has recently been reported, allowing recognition of TAA-loaded cancer cells by specific effector CD4^+^ T cells. The generation of tumor-reactive cytotoxic T lymphocytes (CTLs) is mostly driven by the antigenicity of the dying tumor cells (44). The capacity of OVs to induce T cells specific for the entire TAA repertoire is an important feature of this therapy. OV-induced tumor cell death and the following epitope spreading in the TME can be seen as a personalized immunotherapeutic approach, without the need for prior identification of the TAA.

Although OV therapy has beneficial effects on the immune system the strength of the induced immune response depends on the particular virus strain that is used, the tumor burden and the immunogenicity. This will determine the outcome of the therapy (45). At this moment the first generation of OVs has been validated in recent clinical trials for their anti-cancer potential (46).

### Radiotherapy

#### Photon and Particle Radiotherapy

In the past century, radiotherapy (RT) has been a strong pillar in the treatment of cancer. Currently, RT is the frontline therapy for approximately 50% of all patients with newly diagnosed cancer, alone or in combination with surgery or chemotherapy (47). Recent advances in RT technologies and approaches have focused on limiting toxicity and on achieving greater therapeutic effectiveness (48). The clinical efficacy of ionizing radiation comes principally from the induction of DNA damage, which can result in tumor cell death. The conventional fractionated regimes used in the clinic are built on four biological processes, called the “4Rs of fractionated radiobiology”: Reoxygenation of hypoxic regions in the tumor, Repopulation of tumor cells, Repair of sublethal damage in normal cells and Redistribution of cells to a cell cycle phase which is more radiosensitive (49). However, Golden and Formenti proposed a fifth R: immune-mediated Rejection of the tumor. The “5th R” is based on preclinical studies that demonstrated an important contribution of RT on the TME and on the induction of anti-tumor immune responses (50). The abscopal effect of RT, originally described by Mole in 1953, is a phenomenon where localized radiation of a tumor results in a response at distant metastatic sites outside of the path of radiation (51). Over the last decade the rare abscopal effect has been reported for several cancers, including melanoma, renal cell carcinoma, breast cancer, hepatocellular carcinoma, and other metastatic solid tumors (52–57).

The immunogenic potential of particle radiation therapy (e.g., proton, carbon-ion, …) has also been investigated by different groups. The main difference between particle radiation and x-rays are the physical properties of the beam. X-rays are absorbed in the tissue, leading to an exponential decay of the radiation dose by increasing depth. In contrast, charged particles lose little energy when they enter the body, when their velocity is high, and most energy deep in the tissue (= Bragg peak). Therefore, charged particle therapy produces a more conformal dose distribution thereby minimizing the area of normal tissue exposed to radiation (58). Moreover, heavy particles have a higher relative biological effectiveness (defined as the ratio of dose of a reference radiation (x-rays or γ-rays) and the dose of a rest radiation that produce the same biological effect) with high linear energy transfer (energy deposited per unit track in the tissue by charged particles) (59, 60).

#### Immune Modulation by RT

Preclinical evidence has demonstrated that tumor targeted RT can stimulate the immune system at least via three distinct mechanisms. First, RT can induce ICD, which leads to the release of neo-antigens. Thereby, RT can improve the recognition and killing of tumor cells by CD8^+^ T cells. Moreover, RT can overcome T cell exclusion from the tumor by promoting the release of chemokines that attract effector T cells to the TME. By surmounting the vascular barrier, T cell infiltration is also facilitated. Moreover, RT can upregulate MHC class I and other components of the antigen processing machinery (61, 62). Anti-tumor immune responses are also improved through the expression of pro-inflammatory cytokines and chemokines, as well as natural killer cell (NK) activating ligands that are produced in response to RT (29, 63–65). In addition, activation of cGAS-dependent and STING-dependent pathways trigger type I IFN signaling in DCs, further strengthening adaptive immune responses in response to RT (29). This shows that RT has the potential to trigger antigen-specific adaptive immunity, but in preclinical models radiation often fails to induce T cell responses to most TAAs (66).

Interestingly, radiation was shown to increase the intracellular peptide pool and induce T cell responses to these peptides. This observation suggests that radiotherapy can selectively boost anti-tumor T cell responses to unique radiation-induced antigenic peptides or tumor-related self-antigens (61). This could be extremely valuable in new strategies to combine radiotherapy and immunotherapy for locally advanced cancers. However, for metastatic diseases, it is unknown whether the different antigenic peptides are shared by the irradiated and non-irradiated metastases. Moreover, radiation has an effect on multiple surface molecules that facilitates recognition of irradiated tumor cells by T cells. Therefore, epitopes present in lower abundance or of low affinity for the TCR may not interact with T cells in the non-irradiated metastasis (67, 68). The presence of multiple antigenic targets, leading to polyvalent T cell responses, on irradiated and non-irradiated tumors may solve the concern about the differential specificity of T cells (69, 70).

Although there are multiple mechanisms by which RT can induce immune activation, for a long time, high-dose radiation was thought to be immune suppressive. The immune suppressive effects of RT can be explained by the fact that different immune cells are very sensitive to radiation and can be eradicated at much lower radiation doses than needed to kill cancer cells. Moreover, the TME also contains different subsets of inhibitory immune cells, including Treg, myeloid-derived suppressor cells and tumor-associated macrophages, that can be activated after RT (71–78) Furthermore, it was shown that RT can increase the expression of PD-L1 on melanoma and glioblastoma cells thereby hampering effecting killing of the tumor cells by cytotoxic T lymphocytes (79). This balance between immune activation and immune suppression caused by RT is nicely reviewed by Wennerberg et al. (80) and Lee et al. (81).

In *in vitro* tumor cell models it has been shown that proton radiation, compared to photon radiation, resulted in a higher translocation of calreticulin thereby increasing the cross-priming of TAA and the sensitivity of the tumor cells to CTL-mediated killing (82). *P*reliminary *in vivo* data suggest that carbon-ion radiation, combined with DC injection, correlated with a better activation of the immune system (83). Clinically, two patients experiencing abscopal responses following carbon ion RT without immunotherapy for recurrent colorectal cancer have been reported. However, the question remains whether these abscopal responses were due to ablative dose delivery afforded by particle therapy, an immunogenic effect secondary to high-LET radiation, or a combination of both (84, 85).

The use of localized RT with the goal to act as an *in situ* vaccine is a promising concept, especially when combined with other immunomodulating modalities (as described in sections Physical therapies and immunomodulatory factors). However, successful induction of antitumor immunity by RT is dependent on the balance of immune suppressive and immune activating signals that are generated by RT, depending on the dose and quality of the radiation.

### Physical Therapies

Different destructive treatments that induce a local acute trauma at the tumor site, thereby inducing the release of TAAs, aim to initiate an innate immune response targeting both the treated lesion as well as distinct lesions. These physical therapies can be combined with classical treatment schedules or other immunomodulating factors, with the aim to enhance anti-tumor immune responses. An overview of these physical treatment modalities is given in Table 1.

**Table 1:** Overview of different physical therapies.

**Physical therapy**	**Advantages**	**Limitations**	**Indications**
1. Photodynamic therapy	^*^Limited invasiveness ^*^Selective cytotoxicity ^*^Complementarity with standard of care treatments ^*^All organs can be targeted	^*^Protocols need to be optimized for every patient and tumor type	Bladder cancer, Carcinomas *in situ*, Superficial tumors
2. Electrochemotherapy	^*^Increased drug levels at the tumor site ^*^Induction of systemic immune response ^*^Complementarity with other immunomodulating therapies ^*^Favorable safety profile ^*^Repeated treatments possible	^*^Protocol need to be adjusted for every tumor type ^*^Choice of electrodes ^*^Tumor size and location can limit the success delayed drug perfusion	Cutaneous tumors, Breast cancer, Pancreatic cancer, Colorectal cancer
3. Hyperthermia	^*^Suitable adjuvant for standard of care treatments	^*^Appropriate energy source ^*^Non-selective tissue heating	Breast tumors, Gastrointestinal tumors, Melanoma, Brain tumors, Sarcomas
4. Tumor-treating fields	^*^Non-invasive anti-tumor effect ^*^Complementarity with standard of care treatments	^*^Adverse events including skin irritations, rash, ulcerations and infections ^*^Mechanism of action not clear ^*^Cost-effectiveness	Glioblastoma

#### Photodynamic Therapy (PDT)

Photodynamic therapy (PDT) or photochemotherapy is based on a reaction between light and a photosensitizer in the presence of oxygen. The combination of these components leads to a photochemical reaction that generates reactive oxygen species (ROS), which causes cell death. The localized acute trauma and oxidative stress induced by PDT, provokes a strong acute inflammatory reaction. Moreover, it has been established that PDT can induce an adaptive immune response, both humoral immunity as well as cell-mediated anti-tumor immunity. Different parameters, such as the treatment regimen, treated area and the type of photosensitizer, can influence the type and the strength of the immune response that is induced.

The major advantages of this technique include: the possibility to target any organ in the body, the limited invasiveness, the selective cytotoxicity toward the tumor and the complementarity with classical treatment modalities, including surgery, chemo-and radiotherapy. However, different parameters need to be defined for every patient and its specific tumor type since these can affect the outcome of the treatment. These parameters include the choice of and dose of the used photosensitizer, the time between administering the photosensitizer and exposure to light, the dosage of total light and its fluence rate and the oxygen concentration present in the tumor.

The first clinical use of PDT for cancer therapy dates back to the late 1970s, when five patients with bladder cancer were treated. From then on, many efforts are made to evaluate the effect of PDT in patients -currently over 400 clinical trials can be found on clinicaltrial.gov. The indications include premalignant conditions (e.g., mucous dysplasia, actinic keratosis (e.g., NCT03643744), carcinomas *in situ* (NCT03638622, NCT03133650, NCT03211078), and superficial tumors (such as superficially growing basal cell carcinomas (NCT02367547, NCT03467789). However, in most of the cases PDT is used in combination with other standard of care therapies (86).

#### Electrochemotherapy (ECT)

Electrochemotherapy (ECT) is based on the local application of electric pulses to deliver chemotherapeutic drugs at the tumor site. This reversible electroporation enhances the drug uptake by increasing the permeability of the cell membrane. Thereby potentiating the cytotoxicity of non-permeant chemotherapeutic drugs, such as bleomycin and cisplatin (87, 88). The cytotoxicity of ECT acts on the whole TME and therefore targets directly the tumor cells as well as the interwoven stromal and endothelial cells lining the tumor microvasculature. The cell death induced in these endothelial cells leads to the abrogation of tumor blood flow thereby impairing the viability of tumor cells surrounding the vessels. This results in a massive release of TAAs inducing a systemic immune reaction. This immune response can be enhanced when ECT is combined with other immunomodulatory factors, improving the antigen presentation and survival of effector T cells, such as IL-2, IL-12, GM-CSF, and TNF-α (88).

ECT is mainly used for the local treatment of accessible cutaneous and subcutaneous metastases (since different types of electrodes can be applied, from plate to needle electrodes). However, there are also some limitations to take into account. Different tissues need to be treated according to different protocols, the choice of the electrodes needs to be adapted in accordance with the size and type of the lesions, tumor size and location can determine the success of ECT and, due to delayed drug perfusion, there can be a decreased drug concentration at the tumor site.

Nevertheless, the use of ECT to treat cutaneous tumors has been proven to be a highly efficient and safe approach and is already widely accepted in clinical routine (89). Due to its simple application, favorable safety profile and the possibility of repetitive treatment, this treatment modality can be used for different tumor types with different histologies (88, 89). It has been shown that frequent administration of ECT led to an increase in the rate of complete remissions in breast cancer patients (90). During the years, efforts are made to extrapolate the ECT treatment of easily accessible lesions to non-superficial tumors. Safety, feasibility and efficacy of ECT in locally advanced pancreatic cancer patients in a phase I/II study (91) and in patients with bone metastasis (92) has already been reported. In the latter phase I/II clinical trial, 56% of the patients showed pain relief and in a few patients necrosis of the metastatic lesion was observed (92). A pilot study in patients with unresectable colorectal liver metastases revealed that 55% of the patient population were complete responders and 45% had a stable disease. Additionally, 80–100% of the treated patients had an overall and progression-free survival at 6 months (89, 93). At the moment ECT is usually applied in a palliative setting for patients with unresectable tumors, but it can also be an effective treatment option in minimally invasive oncologic treatments.

#### Hyperthermia

Hyperthermia can be defined as a treatment in which the target tissue, the tumor, is exposed to high temperature. Hyperthermia can be divided into thermal ablation, where the tumor tissue is destroyed directly, or thermal sensitization where the tumor is rendered more susceptible to other treatments (94). Thermal sensitization (40 – 45°C) is most used in the clinic and serves as adjuvant for standard of care treatments like chemotherapy and radiotherapy (95, 96). An elevation in temperature causes tissue changes in the vascular permeability, increase in blood flow and eventually leads to tumor oxygenation.

Combinational strategies with radiotherapy or chemotherapy and hyperthermia have shown clinical benefit for the treatment of a wide range of cancers including breast cancer, gastrointestinal tumors, gynecological tumors, brain tumors, lung tumors, melanomas, and sarcomas (97). Although hyperthermia continues to show clinical benefits in randomized trials, widespread application remains omitted.

One of the challenging issues for hyperthermia is the appropriate means for heat delivery. At this moment four different energy sources can be used: microwave, radiofrequency, laser and ultrasound. In conventional local hyperthermia, the heating happens from the outside-in, which can lead to serious side effects through non-selectivity in tissue heating. Alternatively, the application of nanoparticles as hyperthermia agents was developed to increase the effectiveness of hyperthermia. Nanoparticle-mediated hyperthermia could help reduce the side effects by employing inside-out hyperthermia (94). There exist four different kinds of nanoparticle-mediated hyperthermia: nano-photo-thermal therapy, nano-magnetic hyperthermia, nano-radio-frequency ablation, and nano-ultrasound hyperthermia. Nano-magnetic hyperthermia is the only and first application of Nanoparticle-mediated hyperthermia that has been introduced in the clinic. The main advantage over conventional hyperthermia is the ability of the magnetic nanoparticles to distribute into the tumor hereby creating a difference in temperature between tumor and healthy tissue (98).

#### Tumor-Treating Fields (TTF)

Tumor-treating fields (TTF) represents a treatment modality designed to deliver alternating electrical fields to a malignant lesion. It concerns a cancer treatment specifically used for brain tumors, especially tested for glioblastoma. Different clinical trials have been performed to assess the benefits of this adjuvant therapy in combination with the standard of care in glioblastoma cancer patients. The EF-14 trial (NCT00916409), the largest multinational trial of TTF therapy, showed that both progression free survival and overall survival were prolonged in glioblastoma patients treated with TTF. Common adverse events are skin irritation, including rash, ulceration and infections (99).

TTF may also be synergistic with immunotherapeutic approaches. TTF have been shown to lead to an aberrant mitotic exit (which can induce ICD), expose CRT on cell surface and decrease tumor volume when combined with an anti-programmed cell death 1 (anti-PD-1) drug (100–104).

However, there still is significant skepticism about the TTF device. Questions about the clear mechanism of action, interpretation of the data from the clinical trials and cost-effectiveness of TFF therapy need to be elucidated (105). As such, more promising clinical data and research will be necessary to convince the physicians to apply TTF as standard treatment (106).

### Immunomodulatory Factors

Through the local administration of growth factors, cytokines, and immunomodulatory molecules, we can enhance all the steps needed to induce an effective anti-tumor immune response and counteract the mechanisms that tumors use to escape immune control, while limiting toxicities associated with the systemic administration of these molecules.

These strategies, which can be used as a stand-alone therapy or in combination with OVs and/or RT, will be discussed in detail in the following section. An overview of these strategies is given in Table 2.

**Table 2:** Overview of the different molecules and strategies used for the *in situ* modulation of the tumor microenvironment.

**Immunomodulating factor**	**Mode of Action**	**Indication**	**References**
**GROWTH FACTORS**			
**1.GM-CSF**	**Increase in the number of DCs in the TME**		
^*^T-VEC (OV)		Melanoma	(107)
^*^JX594 (OV)		Melanoma, Liver carcinoma	(108–112)
^*^Combined with RT		Lung carcinoma, Hepatocellular carcinoma	NCT02946138, NCT03113851
**2. FLT3L**	**Increase the mobilization of DCs**	
^*^Combined with chemotherapy		Preclinical	(113)
^*^Combined with RT		Low-grade B cell lymphoma	NCT01976585
**CYTOKINES**			
**1. IL-12**	**Polarization of type 1 helper T Increased IFNγ production by CTLs**		
^*^Systemic delivery		Melanoma, Renal cell carcinoma, Colon carcinoma	(114, 115)
^*^Encapsulated into nanoparticles		Preclinical Ovarian cancer	(116) (117)
^*^Gene electrotransfer		Triple Negative Breast Cancer, Lymphoma, Merkel cell carcinoma, Melanoma	NCT02531425, NCT01579318, NCT0144081
^*^Viruses expressing IL-12		Preclinical	(118–120)
**2. IL-2**	**Expansion and differentiation of effector lymphocytes**		
^*^Systemic delivery		Renal cell carcinoma, Melanoma	(121, 122)
^*^Encapsulated into nanoparticles		Renal cell carcinoma, Melanoma	(123–125)
^*^Combined with α-CTLA-4		Melanoma	NCT01480323, NCT01672450
^*^Combined with RT		Renal cell carcinoma, Melanoma, Non-small cell lung cancer	NCT01884961, NCT02306954, NCT030226236, NCT03224871
**3. TGF-β (blocking)**	**Associated with immunosuppression in the TME**		
^*^Combined with RT		Non-small cell lung cancer, Rectal cancer, Hepatocellular carcinoma, Solid tumors	NCT02581787, NCT02688712, NCT02906397, NCT02937272
**IMMUNOMODULATORY FACTORS**			
**1. Checkpoint inhibitors**	**Releasing the brakes on the immune system and promote function and survival of T cells**		
^*^Systemic delivery		Melanoma, Renal cell carcinoma	Different agents already FDA approved
^*^Combined with OVs		Preclinical Melanoma	(126–131) NCT02263508
^*^Combined with RT		Preclinical, >100 trials in different Solid tumors	(132–139)
**2. CD40 agonist**	**Initiation and propagation of adaptive immune responses**		
^*^Monoclonal antibodies		Preclinical Solid tumors	(140, 141) NCT02379741
^*^mRNA		Preclinical	(142)
^*^Combined with OVs		Preclinical	(143, 144)
**3. OX-40 agonist**	**Delivering co-stimulatory signals to T cells needed for their full activation**		
^*^mRNA		Solid tumors, Lymphoma	NCT03323398
^*^Combined with checkpoint inhibitors		Preclinical	(145)
^*^Combined with OVs		Preclinical	(146–148)
^*^Combined with RT		Prostate cancer, Breast cancer, B cell Non-Hodgkin lymphoma	NCT01642290 NCT03410901
**4. TLR agonist**	**Activation of APCs**		
^*^Monotherapy		Advanced solid tumors, Prostate cancer, Basal cell carcinoma	NCT01984892, NCT03262103, NCT0066872,
^*^Combined with OVs		
^*^Combined with RT		B cell lymphoma, Merkel cell carcinoma, Solid tumors, T cell lymphoma	NCT01976585, NCT02501473, NCT02556463, NCT0088058, NCT02927964
**5. STING agonists**	**Activation of the innate immune system through upregulation of IFNs**		
^*^Monotherapy		Solid tumors, Lymphomas	NCT03172936
^*^Combined with checkpoint inhibitors		Solid tumors, Lymphomas	NCT02675439

#### Growth Factors

Immune responses against malignant cells can be improved by increasing the number of APCs in the tumor that can cross-present TAAs to CD8^+^ T cells (149).

##### Granulocyte macrophage—colony stimulating factor (GM-CSF)

GM-CSF plays an important role in DC recruitment and maturation but also facilitates the homing of CTLs in the TME. Multiple vaccine platforms include GM-CSF in their formulations and the goal of administering it intratumorally is to increase the number of DCs in the TME (149, 150). In different preclinical studies it was shown that the IT expression of GM-CSF resulted in an effective anti-tumor immune response (151, 152). In patients with melanoma, IT or peritumoral injection of recombinant GM-CSF results in an increase in the number of DCs in treated tumor lesions but this did not always result in better anti-tumor responses and effects on progression free survival (149, 153–155). A current phase I study investigates the IT administration of GM-CSF in pancreatic cancer patients (NCT00600002).

Although GM-CSF has therapeutic potential as a monotherapy, combinations with other immune modulating agents, such as OVs or radiotherapy, might potentiate the effects (149). Using OVs engineered to express cytokines to increase the number of APCs at the tumor site is also a solid strategy to enhance the anti-tumor effect of OVs. T-VEC, an attenuated herpes simplex virus incorporating a GM-CSF transgene, was granted marketing approval by FDA and EMA in 2015 for IT therapy in patients with unresectable stage 3 and 4 melanoma (107). Similar a vaccinia virus engineered to express GM-CSF, JX-594, has been shown to selectively target and replicate in tumor cells and has anti-tumor efficacy in both a preclinical and clinical setting (108). IT delivery of JX-594 is well tolerated in patients with liver cancer and melanoma, resulting in encouraging effects on the survival and overall response in both treated and untreated lesions (109–112). The combination of recombinant GM-CSF and RT is currently being evaluated in 5 phase II clinical trials in metastatic lung cancer and hepatocellular carcinoma.

##### Fms-related tyrosine kinase 3 ligand (Flt3L)

Flt3L is a key growth factor in the generation of DCs from hematopoietic progenitors present in the bone marrow (149, 156). Subcutaneous and systemic injection of Flt3L has proven to stimulate mobilization of different subsets of DCs to the peripheral blood of both healthy donors and patients with melanoma or colon cancer (157, 158).

Vaccination with Flt3L prior to tumor challenge has shown to be able to prevent tumor growth in mouse models of colon cancer and leukemia, however the therapeutic administration of Flt3L could not cure already established tumors. In contrast, IT administration of an adenovirus expressing Flt3L together with systemic chemotherapy induced complete remission of established murine hepatoma and colon cancer (113).

Systemic Flt3L combined with RT led to a significant growth delay of both the irradiated tumor and the non-irradiated tumor compared to the non-treated control groups. This abscopal effect was dependent on the induction and activation of T cells (159). Currently, one clinical trial is testing the combination of IT Flt3L and poly-ICLC with low dose RT in low-grade B-cell lymphoma patients (NCT01976585). This study reported partial and complete remissions of both treated and untreated lesions associated with increased DC numbers (160).

#### Cytokines

Cytokines are potent immune modulating proteins with an important role in the maintenance of immune homeostasis, initiation, and regulation of inflammatory responses, controlling pathogens and enforcing tolerogenic mechanisms. The *in situ* delivery of cytokines represents an attractive approach to remodel the immune system and their adjuvant properties can increase vaccine efficacy (123).

##### Interleukin-12 (IL-12)

IL-12 is a cytokine that plays a major role in the regulation of adaptive T cell responses. Various immune cell types—but particularly myeloid APCs—secrete IL-12 in response to infection or inflammation. IL-12 secretion leads to the polarization of type 1 helper T (Th1) cells and an increase in the activity and IFNγ production of CTLs, stimulating them to kill infected cells or tumor cells (123, 149).

The systemic delivery of IL-12 has been tested in melanoma, renal cell carcinoma and colon carcinoma patients, but unfortunately several patients experienced considerable hepatic and hematologic toxicity and only a modest anti-tumor efficacy could be observed (114, 115). In contrast, the IT administration of IL-12 is correlated with less toxicity and different methods are being evaluated in order to deliver IL-12 locally (149).

One approach is the use of particle-encapsulated cytokines in order to deliver the cargo in a specific (to certain cell types and tissues) and protected manner. IT administration of IL-12 encapsulated into polymer microspheres induces the regression of primary and metastatic murine lesions (116). These cytokine depots have shown their potential for anti-cancer therapies, but the challenge remains to translate their preclinical promise into a clinical application (123). The intra- or peritumoral use of a lipopolymer formulated human IL-12 plasmid has been tested in an early study including 13 ovarian cancer patients. An increase in IL-12 and IFNγ levels could be detected in peritoneal fluid (but not serum) and a minority of patients showed treatment-related decreases in serum levels of the tumormarker Cancer Antigen-125 (CA-125) (117).

Kamensek et al. tested the IT gene electrotransfer of TNF-α combined with IL-12 in murine melanoma tumors. This approach was proven feasible and effective in eliciting a potent and durable anti-tumor response, resulting in a delayed tumor growth and prolonged survival (161). This delivery method also found its way toward the clinic for the treatment of different cancer types including Triple Negative Breast Cancer (NCT02531425), lymphoma (NCT01579318), and Merkel cell carcinoma (NCT01440816), and the therapy induces objective systemic tumor responses in a significant number of melanoma patients (162).

Different preclinical studies using modified viruses expressing IL-12 resulted in strong anti-tumor immune responses associated with delayed tumor growth and increased survival in various murine cancer models (118–120).

##### Interleukin-2 (IL-2)

IL-2 is one of the most intensively studied cytokines in cancer immunotherapies, because of its important role in the development of an adaptive immune response. It has a wide spectrum of effects on the immune system including the expansion and differentiation of effector lymphocytes—crucial for the development of a specific anti-tumor response.

IL-2 is already approved by the FDA as a first-line treatment for patients with renal cell carcinoma and melanoma, although the systemic administration is associated with significant toxicity. To limit these toxicities, *in situ* delivery of soluble IL-2 has already been tested in a preclinical setting and resulted in the increased infiltration of CD8^+^ T cells and reduced tumor growth in tumor bearing mice (121, 122).

Moreover, the IT injection of IL-2 encapsulated in polymeric microparticles for the treatment of brain or liver tumors, had better results than the use of modified tumor cells expressing IL-2 (123–125). Combining the IT injection of microparticles encapsulating IL-2 with microwave coagulation—to induce tumor cell death—resulted in a systemic tumor-specific immune response in mice bearing lung or hepatocellular carcinomas. These encouraging preclinical observations were extrapolated and tested in the clinic. Patients with renal cell carcinoma or melanoma who received IT treatment with either recombinant IL-2 or IL-2 encoding plasmids suffered from less toxicity (compared to systemic administration) and promising anti-tumor efficacy was observed. Although, treatment of renal cell carcinoma patients with an IL-2 encoding plasmid led to a low number of responses (163, 164), injection of recombinant IL-2 into melanoma metastases induced high response rates resulting in tumor regression. However, IT administration of one lesion failed to cause complete regression of untreated melanoma lesions and was not able to prevent the occurrence of metastases, indicating that the induced immune responses are not strong enough to result in an abscopal effect or to induce long-lasting memory responses (149, 165–167).

Different strategies combining IL-2 with other treatment modalities are heavily being investigated. The IT delivery of IL-2 together with the checkpoint inhibitor anti-CTLA-4, delivered either systemically or locally, represents a promising approach in melanoma patients (NCT01480323, NCT01672450). Preclinical data indicates that the use of TILT-123, a modified adenovirus expressing TNF-α and IL-2, in combination with checkpoint inhibitor or TIL therapy could be an effective treatment. The first phase I trial is planned in patients with advanced melanoma (168, 169). Moreover, different phase I and II studies investigating the combination of IL-2 and RT in renal cell carcinoma, melanoma and non-small cell lung cancer are ongoing (NCT01884961, NCT02306954, NCT030226236, NCT03224871).

##### Transforming growth factor-beta (TGF-β)

Inhibition of immunosuppression mediated by different soluble factors secreted by both the tumor cells and different immunosuppressive cell types infiltrating the TME can convert a “cold” tumor into a “hot” tumor. A known immunosuppressive cytokine that is often released after RT is TGF-β (66, 170, 171).

Preclinical studies have already investigated the effect of inhibiting TGF-β during and after RT and showed that this allows T cells to recognize multiple TAAs leading to a broad immune-mediated regression of both the irradiated tumor and the non-irradiated lesions (66). Currently, different clinical trials are ongoing where TGF-β inhibitors are combined with radiotherapy. Fresolimumab is being tested in the SABR-ATAC phase I/II trial in patients with stage Ia/Ib non-small cell lung cancer (NCT02581787). Two phase I studies are testing Galunisertib in rectal cancer and advanced hepatocellular carcinoma in combination with chemotherapy and RT (50.4–54 Gy in 1.8 Gy daily fractions; NCT02688712, NCT02906397). A phase I trial is testing LY3200882 and LY3300054 in combination with chemoradiotherapy in solid tumors (NCT02937272).

#### Immunomodulatory Molecules

In addition to the initial interaction between the TCR and MHC-molecules on APCs, costimulation of the T cells is crucial in order to develop an effective anti-tumor immune response. Different strategies can be envisaged to strengthen the costimulatory signals and prevent downregulation of these interactions in the TME.

##### Checkpoint inhibitors

To prevent auto-immunity and to control immune responses against self-antigens, inhibitory immune checkpoints are expressed on T cells. Currently approved checkpoint inhibitors target the molecules cytotoxic T-lymphocyte-associated protein 4 (CTLA-4), PD-1, and PD-L1. These molecules play a key role in the regulation of immune responses and their expression is often dysregulated in the TME (both on tumor cells and immune cells) thereby preventing effective killing of the tumor cells by effector T cells. CTLA-4 blockade causes a broad enhancement of immune responses and the systemic delivery of anti-CTLA-4 blocking antibodies is currently FDA approved for the treatment of melanoma and renal cell carcinoma. However, the clinical success is hampered by dose-limiting toxicities and immune-related adverse events. Therefore, the IT administration of these checkpoint inhibitors is attractive. Most research is performed on the IT delivery of anti-CTLA-4 (since this was the first checkpoint inhibitor to be approved and is associated with higher toxicities then anti-PD-1/PD-L1).

The use of the slow-release agent Montanide ISA-51 to inject an anti-CTLA-4 antibody peritumorally resulted in local anti-tumor CD8^+^ T cell activation and tumor eradication associated with thousand-fold lower serum levels of antibody compared to the systemic delivery—reducing the adverse events and the risk of auto-immunity (172).

OVs are ideal candidates to combine with monoclonal antibodies against inhibitory immune checkpoints. The IT injection of Newcastle disease virus combined with systemic injection of an anti-CTLA-4 antibody resulted in slower tumor growth, prolonged survival and protected the mice from a subsequent tumor rechallenge in a melanoma setting (126). The combination of T-VEC with ipilimumab was evaluated in a phase Ib study and showed a tolerable safety profile, with a greater efficacy of the combination compared to monotherapy with the single agents (127). More recently, preliminary data from an ongoing phase Ib trial (NCT02263508) showed a response rate in 62% of the treated melanoma patients with combination therapy of T-VEC and pembrolizumab (an anti-PD-1 antibody) (128). Moreover, oncolytic adenoviruses can be engineered to express blocking antibodies against CTLA-4. IT treatment with these viruses results in much higher concentrations of the antibody detected in the TME compared to the serum of mice, with the average plasma concentration staying below the limit that is well-tolerated in humans (129). Also other studies showed that treatment with attenuated viruses expressing blocking antibodies of CTLA-4 resulted in a delayed tumor growth and prolonged survival in murine models of both melanoma and lung cancer. Moreover, treatment with a combination of viruses expressing either an anti-CTLA-4 blocking antibody or GM-CSF resulted in complete tumor regression (130, 131).

Synergy between checkpoint inhibitors and radiation has been demonstrated in different preclinical tumor models, but at this moment the optimal timing of the treatment modalities, the dose, and fractionation regimen of the radiation, resulting in the highest responses are not yet clear warranting further research (69, 132–137). More than 100 clinical trials are currently testing the combinations of different checkpoint inhibitors with different radiotherapy regimens and preliminary data shows that there may be clinical benefit of the combination therapy in cancer patients (137–139).

##### CD40

CD40 is expressed by B cells, professional APCs, as well as non-immune cells and tumor cells. Under inflammatory conditions, CD40 ligand (CD40L) is transiently expressed on T cells and other non-immune cells, and binding to CD40 initiates a variety of molecular and cellular processes including the initiation and progression of cellular and humoral adaptive immunity (173).

Peritumoral injection of a slow-release formulation containing an agonistic anti-CD40 antibody was tested in preclinical tumor models and this treatment resulted in systemic tumor-specific CTL expansion and eradication of distant tumors (140). Another research group molecularly engineered an agonistic antibody with high affinity for CD40 (ADC-1013) and tested its effect in two different bladder cancer models. The IT administration of this immunostimulatory antibody resulted in a long-lasting anti-tumor response and immunological memory (141). A phase I clinical trial evaluating the safety and feasibility of the IT administration in patients with advanced solid tumors is already completed (NCT02379741).

mRNA vaccines can also be used to deliver activation stimuli in addition to TAAs to DCs. TriMix is a mix of three mRNA's encoding for a constitutive active form of TLR4 (caTLR4), CD40L, and CD70. The IT delivery of this mRNA mix (in various mouse cancer models) resulted in systemic therapeutic anti-tumor immunity. In addition, TriMix stimulated anti-tumor T cell responses to spontaneously recognized and internalized TAAs, including a neo-epitope (142).

Oncolytic adenoviruses expressing CD40L have been shown to induce significant anti-tumor effects in mice and patients (143, 144).

##### OX40 and CD137

OX40 and CD137 (4-1BB) are both members of the tumor-necrosis factor receptor superfamily, and are expressed on T cells, including TILs, as well as other immune cell subsets. Ligation of these receptors with their ligands delivers a costimulatory signal to T cells, necessary for their full activation. Targeting of both receptors has been assessed in early clinical trials and shows promising anti-tumor effects (145).

Two anti-CD137 monoclonal antibodies are currently in the clinic: Urelumab (Bristol-Myers Squibb) and PF-05082566 (Pfizer) (174). Unfortunately, Urelumab induced liver toxicity requiring dose reduction for subsequent trials and therefore the drug is now tested in different combination strategies but no longer as a monotherapy (145, 174). In contrast, PF-05082566 was not associated with any dose-limiting toxicities and is also under further investigation in combination with other immunomodulatory therapies (174).

A phase I clinical trial is ongoing where mRNA encoding for OX40 Ligand (OX40L) is intratumorally delivered in patients with refractory solid malignancies or lymphomas (NCT03323398). The anti-tumor effects of a mixture of mRNA molecules encoding for OX40L, IL-23, and IL-36γ in different mouse models after IT injection, either alone or in combination with checkpoint inhibitors is also being tested. Hebb et al. tested whether targeting both CD137 and OX40, in combination with the immune checkpoint inhibitor anti-CTLA-4, could result in a synergistic effect on tumor growth control and survival compared to the targeting of only one receptor. The triple combination administered intratumorally at low doses to one tumor had dramatic local and systemic anti-tumor efficacy in preclinical tumor models. Moreover, the IT administration resulted in superior local and distant tumor growth control, compared to the systemic delivery of the combination (145).

Targeting OX40 and 4-1BB with modified OVs has already proven their promise in preclinical mouse models and will soon be tested in a clinical setting (146, 147). In preclinical studies the use of OX40 led to an enhancement of T cell memory and proliferation, in combination with a suppression of Treg function showing the potential for combining OX40 agonists with RT, surgery or systemic agents (148). A phase I and a phase I/II clinical trial testing an agonistic antibody against OX40 with cyclophosphamide and single fraction RT in metastatic prostate cancer (NCT01642290) and a OX40 agonist (MEDI6469) with different doses SBRT in metastatic breast cancer are currently active. A phase I clinical trial combining an anti-OX40 antibody (BMS-986178) with a TLR9 agonist (SD-101) and RT is tested in patients with low-grade B-cell Non-Hodgkin lymphomas (NCT03410901). This approach envisions the inhibition of tumor cell growth using the TLR9 agonist, activation of T cells by the anti-OX40 antibody and supplementary killing of cancer cells by radiation making them more visible for the immune system.

##### TLR Agonists

*TLR2*. Already 100 years ago William Coley injected Coley's toxins locally in the tumor resulting in regression of sarcoma. These data are translated in the use of Bacillus Calmette-Guérin (BCG) for the treatment of superficial urothelial carcinoma (175). BCG activates TLR2 and TLR4 in macrophages and DCs. This vaccine was primarily developed for the prevention of tuberculosis and is nowadays the standard treatment for patients with *in situ* or non-muscle invasive bladder cancer (176). The IT injection of a genetically engineered, lethal-toxin deficient strain of *Clostridium novyi*, that activates DCs via TLR2, can induce CD8^+^ T cell mediated anti-tumor effects in preclinical renal cell carcinoma, colon carcinoma, and anaplastic squamous cell carcinoma models (177).

*TLR3*. A danger signal that is detected by endosomal TLR3 and the intracellular sensors RIG-I and MDA-5 is dsRNA (149). The IT delivery of poly-ICLC or Hiltonol, a synthetic analog of dsRNA, has already shown its potential in the clinic and a sequential treatment scheme of IT and intramuscular (IM) delivery of poly-ICLC was given to a young male patient with advanced facial embryonal rhabdomyosarcoma with extension to the brain. After treatment, the patient showed tumor inflammation, followed by gradual, marked tumor regression, with extended survival (178). Such results have prompted a phase II clinical trial (NCT01984892) in patients with advanced solid tumors receiving IT poly-ICLC to prime the immune system followed by IM poly-ICLC injections to boost the response. The idea is that these IT/IM booster injections will mimic a viral infection that will result in the release of TAAs upon IT injection and a strong activation of the immune response against these TAAs upon IM injection. Hiltonol is currently intratumorally tested in a phase I neoadjuvant setting in prostate cancer patients (NCT03262103). A phase I/II clinical trial combining IT Flt3L (CDX-301), Hiltonol and low-dose radiotherapy in B-cell lymphoma patients is ongoing (NCT01976585).

*TLR4*. In different transplantable murine tumor models it has been shown that IT treatment with TLR4 agonists, such as lipopolysaccharide (LPS) and monophosphoryl lipid A (MPL A), induces an anti-tumor immune response leading to regression of the tumor. In humans, the IT delivery of the synthetic TLR4 agonist Glucopyranosyl Lipid A (G100) has showed success in early clinical trials in eliciting Th1 polarized anti-tumor immunity in Merkel cell carcinoma and soft tissue sarcoma, in combination with RT (NCT02501473) (175, 176).

*TLR7/8*. Stimulation of TLR7/8 with ssRNA, significantly improves DC maturation, Th1 mediated immunity, cross-presentation of TAAs and humoral immune responses.

Imiquimod is an FDA approved small molecule TLR7/8 agonist, formulated as a dermal cream, for HPV mediated external genital warts, superficial basal cell carcinoma and actinic keratosis. Local imiquimod has been used successfully in immunotherapy combinations to treat transplantable mouse models (179, 180), and was tested in a randomized controlled trial (NCT0066872) in patients with nodular and superficial basal cell carcinoma and demonstrated to be superior to excision surgery. Currently imiquimod is tested in more than 100 clinical trials either alone or in combination with classical treatment modalities (150, 175, 176). Topical application of imiquimod resulted in histological regression in melanoma, superficial breast cancer metastases and in anti-tumor effects in T cell and B cell lymphomas (181–189). Promising abscopal effects could be seen after the topical administration of imiquimod in combination with local RT in a breast cancer mouse model. The treatment resulted in complete regression of locally treated tumors and inhibited tumor growth at untreated sites. This anti-tumor response is dependent on CD8+ T cells and an increase of T cell infiltration was noticed in the tumor lesions (149). The established anti-tumor effect could be augmented by pre-treatment with low-dose cyclophosphamide. This resulted in a protection from tumor rechallenge, suggesting that a long-term memory response against the tumor was induced in mice (180).

Another promising lipid-modified imidazoquinoline is 3M-052. It is evaluated as an adjuvant in many vaccine models and showed promising preclinical results in mouse melanoma and prostate tumor models. Moreover, the anti-tumor effect seen in melanoma mouse models was enhanced by concomitant CTLA-4 and PD-L1 blockade (149, 150, 175, 176, 190). Currently, a new TLR7/8 agonist, MEDI9197, is tested in the clinic. In this phase I study this agonist is delivered by IT injection to patients with solid tumors or cutaneous T cell lymphoma in combination with durvalumab and/or palliative radiation (NCT02556463).

*TLR9*. Bacterial DNA is sensed through the presence of unmethylated CpG motifs by endosomal TLR9. When CpG oligonucleotides were injected IT into human lymphoma lesions objective clinical responses were observed when combined with low-dose limited field RT (NCT00880581) (175, 191–193). Other combinatorial approaches are tested in the clinic in lymphoma patients; such as a phase I/II study combining SD-101, a TLR9 agonist in combination with ipilimumab (NCT02254772), a phase I trial combining anti-OX40 antibody (BMS-986178) together with SD-101 and RT (NCT03410901) and a phase Ib/II trial combining SD-101, ibrutinib and RT (NCT02927964). Treatment is generally well-tolerated, with a dose-related incidence of injection site reactions (149). Raykov et al. demonstrated that the oncolytic parvovirus H-1P enriched for CpG motifs can be used as an anti-tumor vaccine in a rat model for metastatic long cancer (194). Similar effects were observed with a CpG-enriched adenovirus used to treat mice bearing lung cancer and in melanoma models (195).

##### STING agonist

Foreign (viral or bacterial) DNA in cells, is processed via cGAS into cyclic dinucleotides, which are ligands for the intracytoplasmic sensor STING. Activation of the STING pathway leads to a cascade of events ultimately resulting in the transcription of pro-inflammatory IFNs and other genes associated with the innate immune system. Therefore, it was hypothesized that the use of STING agonists could promote an anti-tumor immune response. This hypothesis is supported by different preclinical studies showing that STING is a key mediator in the induction of a T cell response against tumors. Moreover, this pathway was shown to play a role in mediating the anti-tumor effects of different checkpoint inhibitors (196).

The first reported STING agonists are the anti-cancer flavonoids FAA, DMXAA and CMA. But, cyclic dinucleotides are more similar to the natural ligand cGAMP. IT injection of cyclic dinucleotides unleashes a powerful and often curative anti-tumor immune response in different transplantable tumor mouse models, with the induction of clear abscopal effects (197). A phase I clinical trial evaluating the IT injection of ADU-S100 in patients with (accessible) solid tumors and lymphomas (NCT03172936) (196) is ongoing. Another phase I trial investigates the anti-tumor effects of the combination of ADU-S100 and ipilimumab in patients with advanced solid tumors and lymphomas (NCT02675439).

Recently, it was demonstrated that radiation-mediated cure of immunogenic tumors is dependent on host STING (29). Therefore, the targeting of the cGAS/STING pathway in combination with RT is being investigated in preclinical models (24, 198, 199).

## General Conclusions and Future Perspectives

The major benefit of immunotherapy is the generation of memory CD8^+^ T cells thereby providing durable protection against metastasis and preventing relapse of the disease. One obvious limitation for *in situ* vaccination is the need to access the tumor for injection. However, modern imaging techniques, such as computed tomography guidance, enable accurate injection of different tumor types even deep within the body. The induction of tumor cell death and DC activation needs to occur simultaneously (in time and place) in order to lead to robust anti-tumor immune responses. By combining RT, OVs or physical therapies with the local delivery of immunomodulatory factors, both can be achieved resulting in potent immune responses. The challenge for *in situ* vaccination is to develop an optimal approach to circumvent local immunosuppression, which is characteristic for tumors, simultaneously resulting in an effective systemic anti-tumor immune response. It is clear that treating a patient with an *in situ* vaccine early in the disease will have the best results since the immune system of patients with metastatic disease will be weaker due to the presence of more immunosuppressive factors. The evaluation of different *in situ* vaccines in early diagnosed patients without evidence of metastatic disease, for example as neoadjuvant therapy prior to surgery, will show the true potential of *in situ* vaccination strategies and combinations for the treatment of cancer patients.

## Author Contributions

HL, WdM, SdM, and SM wrote sections of the manuscript. MD and KT performed a thorough review of the manuscript adding suggestions for papers to include in the manuscript. All authors contributed to manuscript revision, read and approved the submitted version.

### Conflict of Interest Statement

The authors declare that the research was conducted in the absence of any commercial or financial relationships that could be construed as a potential conflict of interest.
